# Tumour-reactive B cells and antibody responses after allogeneic haematopoietic cell transplantation

**DOI:** 10.1016/j.iotech.2020.07.002

**Published:** 2020-07-23

**Authors:** G. de Jong, M.A. Gillissen, H. Spits, M.D. Hazenberg

**Affiliations:** 1 Department of Haematology, Amsterdam UMC, Amsterdam, The Netherlands; 2 AIMM Therapeutics, Amsterdam, The Netherlands; 3 Cancer Center Amsterdam and Amsterdam Infection and Immunity Institute, Amsterdam, The Netherlands; 4 Department of Experimental Immunology, Amsterdam UMC, Amsterdam, The Netherlands; 5 Department of Experimental Immunohematology, Sanquin Research, Amsterdam, The Netherlands

**Keywords:** B lymphocytes, tumor specific antibodies, immunotherapy, allogeneic hematopoietic stem cell transplantation, graft versus leukemia response

## Abstract

For many high-risk haematologic malignancies, such as acute myeloid leukaemia, the success of therapy relies mainly on invoking a curative antitumour immune response. This can be achieved by inducing a graft-versus-leukaemia response following allogeneic haematopoietic cell transplantation. While the contribution of T cells and natural killer cells to graft-versus-leukaemia responses is established, the contribution of B cells and antibodies is relatively unexplored. This article reviews what is known about the contribution of B cells and tumour-specific antibody responses to a successful graft-versus-leukaemia response leading to eradication of the tumour.

## Highlights

• B cells play a role in graft-versus-leukaemia (GvL) responses after allogeneic haematopoietic cell transplantation.
• Targets of GvL B-cell responses are not unique to individual patients.
• Tumour antigens are shared between haematologic and non-haematologic tumours.
• GvL antibodies may be employed for the development of novel immunotherapies.

## Introduction

Chemotherapy is often effective in eradicating the bulk of the tumour mass of acute myeloid leukaemia (AML), but persistent complete remission of AML can only be achieved when allogeneic haematopoietic stem cell transplantation (HCT) induces a graft-versus-leukaemia (GvL) response that eliminates residual malignant cells.

The importance of allogeneic HCT for the treatment of AML and other haematologic malignancies has been acknowledged since the 1990s, when it became clear that the curative effect of allogeneic HCT relies on the induction of a donor immune response against the recipient’s tumour.^1^ The first successful HCT in humans had been performed decades earlier, in the late 1950s, in two girls with acute leukaemia who received bone marrow from their identical twin sisters.^2^ After having received supralethal, myeloablative total body irradiation in an attempt to eradicate all leukaemic cells, haematopoiesis was restored successfully. However, in both patients, acute leukaemia relapsed within weeks, suggesting that ‘evidently something more than radiation is needed to eradicate leukaemia’.^2^ In the following years, clinical observations supported this hypothesis. It was found that allogeneic HCT, but not autologous HCT, was associated with a reduction in disease relapse, particularly in patients with graft-versus-host disease (GvHD),^1^^,^^3^ suggesting that allogeneic immune responses were protective against disease relapse. In a large cohort of patients, Horowitz et al. confirmed the occurrence of GvL immunity by demonstrating that patients who received syngeneic or T-cell-depleted grafts did not have GvHD and had much higher leukaemia relapse rates.^1^ Additional support for the phenomenon of GvL immunity came from studies on donor lymphocyte infusions (DLIs) in patients with chronic myeloid leukaemia (CML). DLIs induced complete remission in high percentages of allogeneic HCT recipients with relapsed CML, and these remissions were persistent in many of the patients (reviewed by Kolb^4^).^4^, ^5^, ^6^, ^7^

Historically, in GvL, attention has predominantly been focused on T cells; this was based on mouse models which demonstrated the importance of CD4+ and CD8+ T cells to the development of GvHD and GvL responses,^8^ and on the observation that higher relapse rates were reported for patients who had received a ‘T-cell-depleted’ allograft.^1^^,^^9^, ^10^, ^11^ Ex vivo T-cell depletion of human allografts is most often achieved by targeting CD52 [alemtuzumab or (Mab)Campath ‘in the bag’] or by positive selection of CD34+ haematopoietic progenitor cells. As both of these approaches deplete B cells, natural killer (NK) cells and other immune cells, as well as T cells,^12^, ^13^, ^14^ relapse after transplantation with an ex vivo T-cell-depleted allograft cannot be attributed solely to the absence of T cells. The contribution of other lymphocyte subsets to the GvL effect has been demonstrated; for example, Ritz et al. were the first to describe the reactivity of NK cells against AML in allogeneic HCT recipients.^15^ In addition, a number of publications have demonstrated the contribution of B cells to the successful immune response against solid tumours.^16^, ^17^, ^18^, ^19^ This article provides an overview of the literature available on B cells in GvL tumour immunology, and places it in the context of potential applications of B cells and tumour-specific antibodies as novel cancer immunotherapies.

## Circulating antibodies and GVL responses

Several studies have addressed the question whether antibodies could be involved in GvL responses. Serum screening against the gene products of a CML-derived cDNA library revealed the appearance of antibodies against CML-associated antigens at the time of clinical responses to DLIs, suggesting functional involvement of these antibodies in GvL immunity. One of the targets identified was the related adhesion focal tyrosine kinase (RAFTK), an intracellular protein expressed in haematopoietic cells.^7^ Other targets included CML28 (hRrp46; a component of the human exosome, a multiprotein complex involved in 3’ RNA processing) and CML66 (a well-conserved protein with unknown function). CML28 and CML66 are expressed abundantly by CML blasts and CD34+ haematopoietic progenitor cells of patients with CML. Patients with relapsed CML who responded well to DLIs produced high-titre CML28-specific immunoglobulin G (IgG) antibodies.^20^^,^^21^ Another serologic screening of a CML cDNA library revealed antibody responses against the receptor for hyaluronan-acid-mediated motility (RHAMM) and the intracellular target M-phase phosphoprotein 11 in patients with CML, and in patients with AML, melanoma, renal cell carcinoma and other malignancies, but not in healthy volunteers or patients with autoimmune diseases.^22^, ^23^, ^24^ The latter indicates that the immune system is also capable of recognizing immunogenic tumour-associated antigens such as RHAMM outside of the allogeneic setting. Finally, protein microarrays were used to screen sera of allogeneic HCT recipients with GvL responses for tumour-reactive antibodies.^25^^,^^26^ Approximately 65% of patients with AML produced antibodies against nuclear and spindle-associated protein 1, an antigen expressed by CD34+CD90+ haematopoietic stem cells. These antibodies were not detected prior to transplantation, nor were they found in healthy volunteers.^26^

GvL antibody responses were also mounted against minor histocompatibility antigens (MiHAs) – polymorphic peptides that are presented on cell surfaces of haematopoietic and non-haematopoietic cells in the context of major histocompatibility complexes (MHCs). Miklos et al. demonstrated that in sex-mismatched male patients undergoing HCT, the presence of antibodies directed against particular, well-characterized MiHAs encoded by genes on the Y chromosome (H-Y antigens), such as dead box RNA helicase Y (DBY), correlated with disease-free survival in a large cohort of over 100 patients.^27^^,^^28^ Not only intracellular or membrane-expressed proteins or protein complexes are targeted by tumour-reactive antibodies. When allogeneic HCT recipients were vaccinated with autologous irradiated AML cells that were engineered to secrete granulocyte-macrophage colony-stimulating factor, a broad antibody response was elicited. Antibodies were directed against intracellular proteins and a number of angiogenic cytokines.^29^ Angiogenesis may play a role in AML pathophysiology: bone marrow microvasculature is increased in patients with AML, AML cells interact with the vascular endothelium and release angiogenic cytokines, and inhibition of these angiogenic cytokines had antileukaemic effects in experimental settings.^30^ The development of antibodies against angiogenic cytokines after vaccination was associated with a better outcome in allogeneic HCT recipients.^29^

## Functional involvement of tumour-specific antibodies in GVL responses

Although the timing of appearance of these antibodies suggests a relationship with the clinical GvL responses observed in these patients, they cannot be taken to prove functional involvement in GvL immunity, particularly because most of these antibodies were targeting intracellular targets. However, two case reports provided evidence for a functional role of B cells in GvL responses. One described a patient who had received an allogeneic HCT for acute lymphoblastic leukaemia (ALL) and who relapsed after receiving treatment with the B-cell-depleting CD20 antibody rituximab for steroid-refractory chronic GvHD.^31^ Our group investigated the B-cell repertoire of a similar patient who had an AML relapse soon after allogeneic HCT.^32^ Rapid tapering of immunosuppressants resulted in strong immune responses inducing complete remission of AML, but also caused severe, steroid-refractory GvHD. Treatment with rituximab resolved GvHD, but AML relapsed soon after. B cells from this patient (of donor origin, as demonstrated by chimerism analysis) were isolated at the time of maximum tumour suppression, before rituximab therapy, and tumour-specific B-cell clones producing antibodies specifically binding to AML blasts were found. One of these antibodies induced complement-mediated lysis of AML cells, suggesting a functional role for this antibody in this patient’s GvL response.^32^

To further investigate whether B cells could be involved in GvL responses, we investigated the B-cell repertoire of three patients with AML who mounted potent tumour-clearing GvL responses after allogeneic HCT.^33^ All patients had B cells that were of HCT donor origin and which produced antibodies that recognized antigens expressed by AML cells. In all three patients, antibodies were identified that target the U5 snRNP200 complex, a large multiprotein complex that is part of the spliceosome. In non-malignant cells, the U5 snRNP200 complex is expressed in the cell nucleus and cytoplasm, but the presence of the U5 snRNP200 complex was demonstrated on the cell membrane of AML blasts in approximately 30% of cases. Strikingly, U5 snRNP200 complex antibodies were cytotoxic: they induced death of AML cells in the absence of effector cells or complement in vitro, and in NSG mice grafted with THP-1 cells in vivo.^33^ In addition, one patient with AMLwas found to produce antibodies against a sialylated epitope on CD43 that is expressed on myeloid but not on B and T cells. CD43sialylated (CD43s) is overexpressed on leukaemic blasts of all World Health Organization 2008 types of AML and myelodysplastic syndrome. AT1413, the antibody that recognizes CD43s, induced death of AML cells via antibody-dependent cellular cytotoxicity (ADCC) and complement-mediated lysis in vitro. In human immune system (HIS) mice grafted with human AML, treatment with AT1413 was highly efficacious in vivo.^34^ In addition, in patients who were treated with allogeneic HCT for relapsed multiple myeloma and who achieved complete remission after DLIs, antibodies were detected that were capable of eliminating myeloma cells.^35^ One of the targets was identified as B-cell maturation antigen (BCMA), a member of the tumour necrosis factor (TNF) superfamily that is expressed by mature B cells. BCMA-expressing myeloma cells were killed via ADCC when incubated with sera from patients responding to DLIs.^36^ The observation that patients with potent graft-versus-tumour (AML, multiple myeloma) responses generated antibodies capable of inducing death of tumour cells suggests the clinical relevance of these antibodies.

B-cell-mediated immune responses can be either dependent or independent of T cells. In a patient who cleared CML after DLI, the appearance of CD4^+^ T cells reactive against a polymorphic MHC-II restricted peptide derived from the enzyme protein kinase 2 beta (PTK2B; also known as RAFTK) coincided with the appearance of antibodies directed against the same target, suggesting that the B-cell response was T-cell dependent in this case.^37^ Similar post-DLI-coordinated T- and B-cell responses were observed against CML66 and the MiHA DBY.^38^^,^^39^ However, details on the phenotype of these helper T cells are lacking. Recently, it was shown that CD4+ T follicular helper (Tfh) cells, which are located in germinal centres in lymph nodes, can promote B-cell immunoglobulin secretion and maturation resulting in chronic GvHD,^40^ but no data on the role of Tfh cells in GvL responses are available.

**Figure 1:**
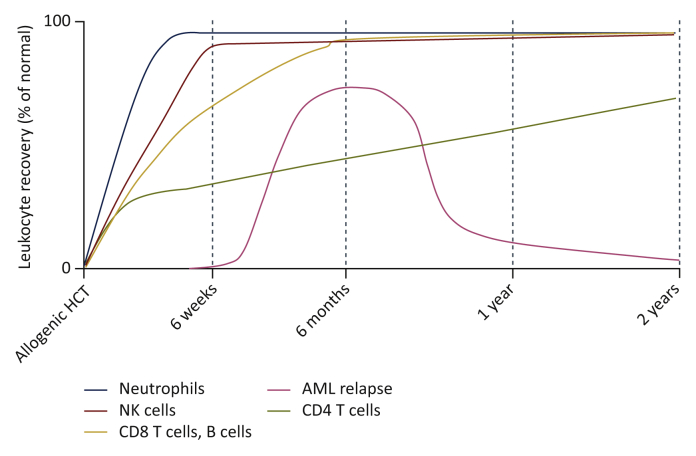
Immune reconstitution and relapse of acute myeloid leukaemia (AML). Immune reconstitution dynamics after allogeneic haematopoietic cell transplantation (HCT) differ between immune cell subsets. Innate cells such as neutrophils and natural killer (NK) cells recover within weeks. Reconstitution of numeric B cells and CD8 T cells occurs within approximately 6 months, although their subset compositions may be altered for much longer. Reconstitution of CD4 T cells, particularly naïve CD4 and naïve CD8 T cells, is slow and often incomplete. The majority of AML relapses occur in the first 6 months after allogeneic HCT, at a time when the adaptive immune system has not yet fully recovered.

It can be questioned whether sufficient numbers of helper T cells are available early after allogeneic HCT. Most AML relapses occur within 6–9 months of allogeneic HCT, suggesting that some of the successful GvL responses are generated at a time when T-cell reconstitution is still incomplete (Figure 1).

Our search for AML-specific antibodies in patients with AML with potent GvL responses, as described above, uncovered many antibodies of interest, most of which were of the IgG3 isotype (and some of the IgG1 isotype).^32^, ^33^, ^34^ This confirms the findings of others, who also found dominance of IgG1 and IgG3 antibodies.^41^ IgG3 isotype antibodies may be generated without the help of T cells.^42^ The predominance of the IgG3 antibodies found may indicate that these antibodies were generated independent of T-cell help. The high-risk nature of AML of these patients entails that the absence of GvL immunity would have been associated with early relapse, and raises the hypothesis that these antibody responses were mounted early after allogeneic HCT, independent of T-cell help, at a time when numbers of reconstituted donor T cells were still low.

## B cells beyond antibodies

**Figure 2:**
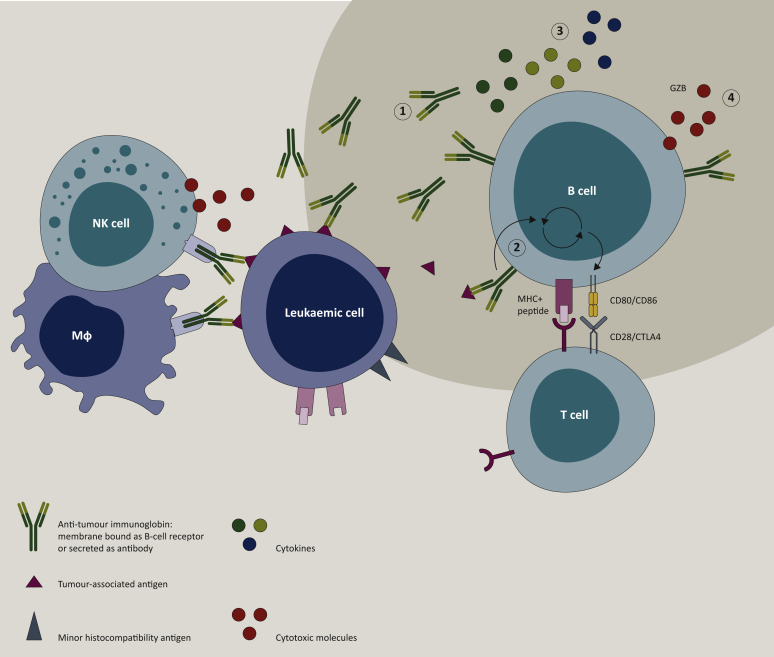
B cells in graft-versus-leukaemia responses. B cells can exert antitumour effects via a number of mechanisms. (1) Antibodies secreted by plasma cells can induce antibody-dependent cellular cytotoxicity, antibody-dependent cellular phagocytosis, complement-dependent cytotoxicity or can be directly cytotoxic. (2) B cells can act as antigen-presenting cells when cognate antigens bound to membrane-expressed immunoglobulin are internalized, processed and presented in the context of major histocompatibility complex (MHC) to T cells. (3) B cells can modulate the tumour microenvironment and antitumour immune responses via the secretion of pro-inflammatory cytokines, such as interleukin (IL)-2, tumour necrosis factor-α, IL-6, IL-12, migration inhibitory factor and interferon-γ. (4) B cells can produce cytotoxic granzyme B (GZB); Mϕ, macrophage.

B cells can develop into plasma cells that produce antibodies, but they can exert a number of other functions that may also contribute to the immune-mediated eradication of tumour cells. B cells can function as antigen-presenting cells by presenting antigen in MHC class II after B-cell-receptor-antigen internalization and processing (Figure 2).^43^, ^44^, ^45^ Vaccination of mice with melanoma or lymphoma with antigen-loaded, CD40-ligand-activated B cells led to tumour-specific T-cell responses and a significant delay in tumour growth.^46^ One study comparing tumour-draining lymph nodes and non-tumour-draining lymph nodes of a small group of patients with solid tumours (of the bladder, colon, skin, pancreas or prostate) found an increased proportion of B-cell plasmablasts in metastatic lymph nodes. They also demonstrated the presence of CD19+ B cells with an activated phenotype, as indicated by CD86 expression, in these lymph nodes, fitting with an antigen-presenting role for B cells in this context.^47^

In various solid tumours, B cells are one of the dominant lymphocyte populations in the tumour microenvironment. They interact with T cells (cytotoxic CD8 T cells, CD4 Tfh cells), frequently organized in tertiary lymphoid structures (TLSs), where they can differentiate into memory B cells, antibody-producing plasma cells or antigen-presenting B cells.^48^, ^49^, ^50^, ^51^, ^52^, ^53^ The presence of TLSs and active B-cell responses have been associated repeatedly with favourable outcomes, also after checkpoint inhibitor therapy.^16^, ^17^, ^18^, ^19^^,^^54^ In addition, B cells can activate or enhance local immune responses by secreting pro-inflammatory cytokines, such as interleukin (IL)-2, TNF-α, IL-6, IL-12, macrophage migration inhibitory factor and interferon-γ, to activate T cells, NK cells and macrophages.^55^ In addition, it has been proposed that B cells have direct cytotoxic effects via their production of granzyme B.^16^^,^^56^ The presence of TLSs and immune modulatory or cytotoxic donor B cells in bone marrow of allogeneic HCT recipients remains to be explored.

## Application of novel tumour-associated antigens for therapy

The remarkable progress in cancer immunotherapy for solid tumours, ALL and lymphomas raised hopes that immunotherapy for AML would be a matter of time. However, this has been a challenge thus far. Targets that are currently under study in AML include CD33, CD38 and CD123 (IL-3 receptor). Similar to targeting CD19 in B-ALL and B-cell lymphomas by chimeric antigen receptor (CAR)-T cells and CD19-directed bispecific T-cell engager (bTCE) therapy, or CD38 in multiple myeloma, these are all antigens that are also expressed by non-malignant haematopoietic progenitor cells (see review of Assi et al.).^57^

Identification of donor-derived antibodies directed against tumour-associated host antigens from patients cured after allogeneic HCT can help with indentifying novel tumour-associated antigens, independent of the context of MHC (Table 1). This seems of particular interest in AML, where downregulation of MHC appeared to be an important mechanism of escape from antileukaemic T-cell immune responses in relapse after allogeneic HCT.^58^ The analyses of B-cell or antibody repertoires in patients with AML who received allogeneic HCT have revealed tumour targets that may be employed for the development of tumour immunotherapies. Patient-derived AML-targeting antibodies may be explored for their capacity to enhance the effect of conventional chemotherapy, similar to the CD20 antibodies rituximab and obinutuzumab that significantly improved the prognosis of patients with B-cell non-Hodgkin lymphoma when applied in conjunction with conventional chemotherapy (R-CHOP, O-CHOP).^59^^,^^60^ AML-targeting antibodies can also be developed as antibody–drug conjugates or as vaccines to induce long-term antitumour T-cell responses. We have recently demonstrated the potency of the CD43s-specific antibody AT1413 in a bTCE format with AT1413 coupled to two T-cell targeting fragments.^61^ AT1413-bTCE was highly effective in inducing T-cell-mediated cytotoxicity of AML cells in vitro, and in HIS mice inoculated with human AML. Despite low-level expression of CD43s on non-malignant human haematopoietic progenitor cells, AT1413-bTCE treatment did not affect normal haematopoiesis.^61^ As described above, antibodies against the B-cell maturation antigen (BCMA) were found in patients with multiple myeloma after allogeneic HCT. A BCMA-specific antibody has been clinically developed as an antibody–drug conjugate and shows promising first results in patients, and BCMA-targeting CAR-T cells have shown clinical effect in a phase I study.^62^, ^63^, ^64^, ^65^ This proves that tumour antigens identified by carefully studying natural antitumour B-cell responses can be successfully employed clinically.

**Table 1:** Tumour antigens with potential for clinical application, including the antibody targets described in the manuscript, the technology used to identify these antibodies and (pre)clinical evaluation

Antigen	Antibody identification	Clinical evaluation	References
Targets of allogeneic B-cell responses			
BCMA	cDNA serum screen of patients with MM after allogeneic HCT and DLI	Antibody–drug conjugate, CAR-T cells	^35^^,^^36^^,^^63^, ^64^, ^65^
CML28 CML66	cDNA serum screen of patients with CML after allogeneic HCT and DLI		^7^^,^^20^^,^^21^^,^^85^
RAFTK (PTK2B)	cDNA serum screen of patients with CML after allogeneic HCT and DLI and western blot analysis of serum specifically for PTK2B		^7^^,^^37^
NuSAP1	Protein array serum screen of patients with AML		^26^
snRNP200	Screening of immortalized donor-derived memory B cells after allogeneic HCT for AML	Tested in mouse models	^33^
CD43s	Screening of immortalized donor-derived memory B cells after allogeneic HCT for AML	Tested in mouse models	^34^^,^^61^^,^^68^
Targets of autologous B-cell responses relevant for haematologic malignancies			
CD9	Screening of immortalized patient-derived memory B cells after adoptive T-cell transfer of tumour-reactive T cells for melanoma	Preclinical evaluation	82, 83, 84^,^^86^, ^87^, ^88^, ^89^
MUC1	Identified in serum of patients with many different types of tumours	Vaccine, CAR-T cells	69, 70, 71, 72, 73, 74NCT04020575

AML, acute myeloid leukaemia; BCMA, B-cell maturation antigen; CAR, chimeric antigen receptor; CML, chronic myeloid leukaemia; DLI, donor lymphocyte infusion; HCT, haematopoietic cell transplantation; MM, multiple myeloma; MUC1, mucin 1; NuSAP1, nuclear and spindle-associated protein 1; RAFTK, related adhesion focal tyrosine kinase.

## Tumour-associated antigens are shared between solid and haematologic cancers

Application of tumour-selective antibodies do not have to be restricted to a specific tumour cell type, as illustrated by daratumumab. This antibody, which targets CD38, was first developed as a therapy for multiple myeloma in which CD38 is overexpressed. Daratumumab is now being evaluated for the treatment of AML and ALL in phase II clinical trials (NCT03067571 and NCT03384654, clinicaltrials.gov). Interestingly, tumour-associated antigens can also be shared between liquid and solid malignancies. CD43, expressed highly on haematopoietic cells, has also been shown to be aberrantly expressed in solid tumours, such as colon and breast cancer.^66^^,^^67^ This suggests that CD43 can also be targeted in solid tumours. We have demonstrated that AT1413 can be used to target CD43s-expressing melanoma cells (De Jong et al., manuscript in preparation).^68^ Another example is the transmembrane glycoprotein mucin 1 (MUC1), which, like CD43, is overexpressed by and differentially glycosylated in a wide range of solid and haematologic tumours.^69^ Antibodies against MUC1 have been detected in the serum of patients with cancer,^70^, ^71^, ^72^ and various clinical trials testing vaccines against MUC1 in patients with multiple myeloma and solid tumours have been performed, albeit with varying degrees of success.^73^^,^^74^ Currently, CAR-T cells directed against MUC1 are being tested in a phase I clinical trial in patients with metastatic breast cancer (NCT04020575). A third example of a tumour-associated antigen expressed on haematologic as well as solid malignancies is CD9. CD9 is a member of the tetraspanin protein family, with broad but not ubiquitous tissue expression (skin, gut epithelium, lung, fibroblasts, bile ducts, neuronal tissue, endothelium, adrenal cortex).^75^^,^^76^ Tetraspanins have a myriad of functions, and play a role in metastasis and tumour progression.^77^ CD9 is overexpressed in precursor B-ALL, AML, glioblastoma, gastric carcinoma and breast cancer.^78^, ^79^, ^80^, ^81^, ^82^ A CD9 antibody-producing B cell was isolated from the B cells of a patient that was cured of stage IV metastatic melanoma after adoptive T-cell therapy (abstract EACR2018).^83^ Multiple CD9-targeting antibodies have been developed, inhibiting growth or irradicating tumours in mice, indicating a potential for CD9 as a therapeutic target (Table 1).^82^^,^^84^

## Concluding remarks

Many studies are now pointing to a significant contribution of B cells and antibodies in successful GvL immunity. Moreover, recent studies indicate that tumour-infiltrating B cells are also beneficial for survival in solid tumours. Studying the role of B cells in tumour immunity contributes to a more comprehensive understanding of antitumour immunity, and will lead to the identification of novel tumour antigens that can be employed to develop novel immunotherapies to treat cancer.
